# Commensals Serve as Natural Barriers to Mammalian Cells during Acanthamoeba castellanii Invasion

**DOI:** 10.1128/Spectrum.00512-21

**Published:** 2021-12-22

**Authors:** Yu-Jen Wang, Chun-Hsien Chen, Jenn-Wei Chen, Wei-Chen Lin

**Affiliations:** a Institute of Basic Medical Sciences, College of Medicine, National Cheng Kung University, Tainan, Taiwan; b Department of Clinical Laboratory, Chest Hospital, Ministry of Health and Welfare, Tainan, Taiwan; c Department of Parasitology, College of Medicine, National Cheng Kung University, Tainan, Taiwan; d Department of Microbiology and Immunology, College of Medicine, National Cheng Kung University, Tainan, Taiwan; Wayne State University

**Keywords:** *Acanthamoeba castellanii*, commensals, mammalian cells, cytotoxicity, opportunistic pathogen

## Abstract

Acanthamoeba castellanii is a free-living, pathogenic ameba found in the soil and water. It invades the body through ulcerated skin, the nasal passages, and eyes and can cause blinding keratitis and granulomatous encephalitis. However, the mechanisms underlying the opportunistic pathogenesis of A. castellanii remain unclear. In this study, we observed that commensal bacteria significantly reduced the cytotoxicity of the ameba on mammalian cells. This effect occurred in the presence of both Gram-positive and Gram-negative commensals. Additionally, commensals mitigated the disruption of cell junctions. *Ex vivo* experiments on mouse eyeballs further showed that the commensals protected the corneal epithelial layer. Together, these findings indicate that A. castellanii is pathogenic to individuals with a dysbiosis of the microbiota at infection sites, further highlighting the role of commensals as a natural barrier during parasite invasion.

**IMPORTANCE** Acanthamoeba castellanii, an opportunistic protozoan widely present in the environment, can cause *Acanthamoeba* keratitis and encephalitis in humans. However, only a few reports describe how the ameba acts as an opportunistic pathogen. Our study showed that the normal microbiota interfered with the cytotoxicity of *Acanthamoeba*, persevered during *Acanthamoeba* invasion, and reduced corneal epithelium peeling in the mouse eyeball model. This suggests that commensals may act as a natural barrier against *Acanthamoeba* invasion. In future, individuals who suffer from *Acanthamoeba* keratitis should be examined for microbiota absence or dysbiosis to reduce the incidence of *Acanthamoeba* infection in clinical settings.

## INTRODUCTION

Acanthamoeba castellanii is a free-living ameba found in a wide variety of environments, indicating its ability to survive under adverse conditions. It feeds on bacteria, fungi, and other protists (1, 2). Obligate intracellular pathogens, such as *Legionella* spp., Mycobacterium avium, *Rickettsia* spp., Chlamydia spp., and giant viruses, can grow within *Acanthamoeba* (3–6). These bacteria adapt to the host environment through gene transfer (7), which, in turn, enhances their evolution and pathogenicity (8, 9). For instance, *Legionella* spp. form a membrane-enclosed microenvironment within A. castellanii via fusion with other membrane-bound vesicles (10). Vibrio cholerae can escape degradation in the cytoplasm of A. castellanii by effectively neutralizing pH changes, digestive enzymes, and the production of reactive oxygen radicals in the ameba (11).

In contrast, bacteria in the human normal microbiota, such as Staphylococcus aureus and Escherichia coli, can serve as food sources for *Acanthamoeba* cells (12). Before being ingested by A. castellanii, bacteria adhere to the ameba’s outer membrane. The surface of A. castellanii expresses a 130-kDa mannose-binding protein (MBP) that plays a role in food source recognition (13). Using the mannose saturation assay, mannose-selected *Acanthamoeba* cells exhibit a significant decrease in E. coli K-12 uptake (14). Polyclonal serum binding to MBP inhibits the association of S. aureus, which suggests that *Acanthamoeba* uses MBP in phagocytosis with S. aureus (15). Further, *Acanthamoeba* can ingest foods into phagosomes and has lysosomal enzymes responsible for digestion and nutrient acquisition (16). However, studies of the influence of diverse microbial ingestion on the pathological microenvironment of A. castellanii are lacking.

*Acanthamoeba* trophozoites can invade humans through the eyes, nasal passages, and ulcerated or broken skin. When *Acanthamoeba* cells enter the eyes, they can cause severe *Acanthamoeba* keratitis (AK) (17). When they enter through the respiratory system or a skin wound, they can invade the central nervous system by hematogenous dissemination, causing granulomatous amebic encephalitis (GAE) or skin lesions in individuals with compromised immune systems (18). Recent investigations have shown that the human ocular microbiota mostly comprises coagulase-negative Staphylococcus, Staphylococcus aureus, and Escherichia coli (19–21). Staphylococcus epidermis, S. aureus, and E. coli have been the focus of many studies on colonization resistance in the skin (22, 23). A study of the nasal microbiota in healthy humans demonstrated that *Corynebacterium* and Staphylococcus were prevalent in most samples (24). Previous studies have shown that the resident microbiota functions to establish and maintain human immune homeostasis (25, 26). Therefore, upon penetration of A. castellanii into the eyes, nasal passage, and skin, the relationship between the ameba and the host microbiota might play a crucial role in the progression of AK and GAE.

In this study, we evaluated interactions between A. castellanii and commensals through the cytopathic effect (CPE) assay and recorded the results of a triculture involving A. castellanii, S. aureus, or E. coli and mammalian cells over time. The processes involved in A. castellanii pathogenicity were examined. Epithelial cell junction proteins were assessed to investigate the influence of A. castellanii on its microenvironment. These results were validated using an *ex vivo* mouse model. Thus, this research will help us better understand the role of normal microbiota and its dysbiosis in AK and GAE progression.

## RESULTS

### Heat-killed commensals reduced *Acanthamoeba* pathogenicity in C6 cells.

**FIG 1:**
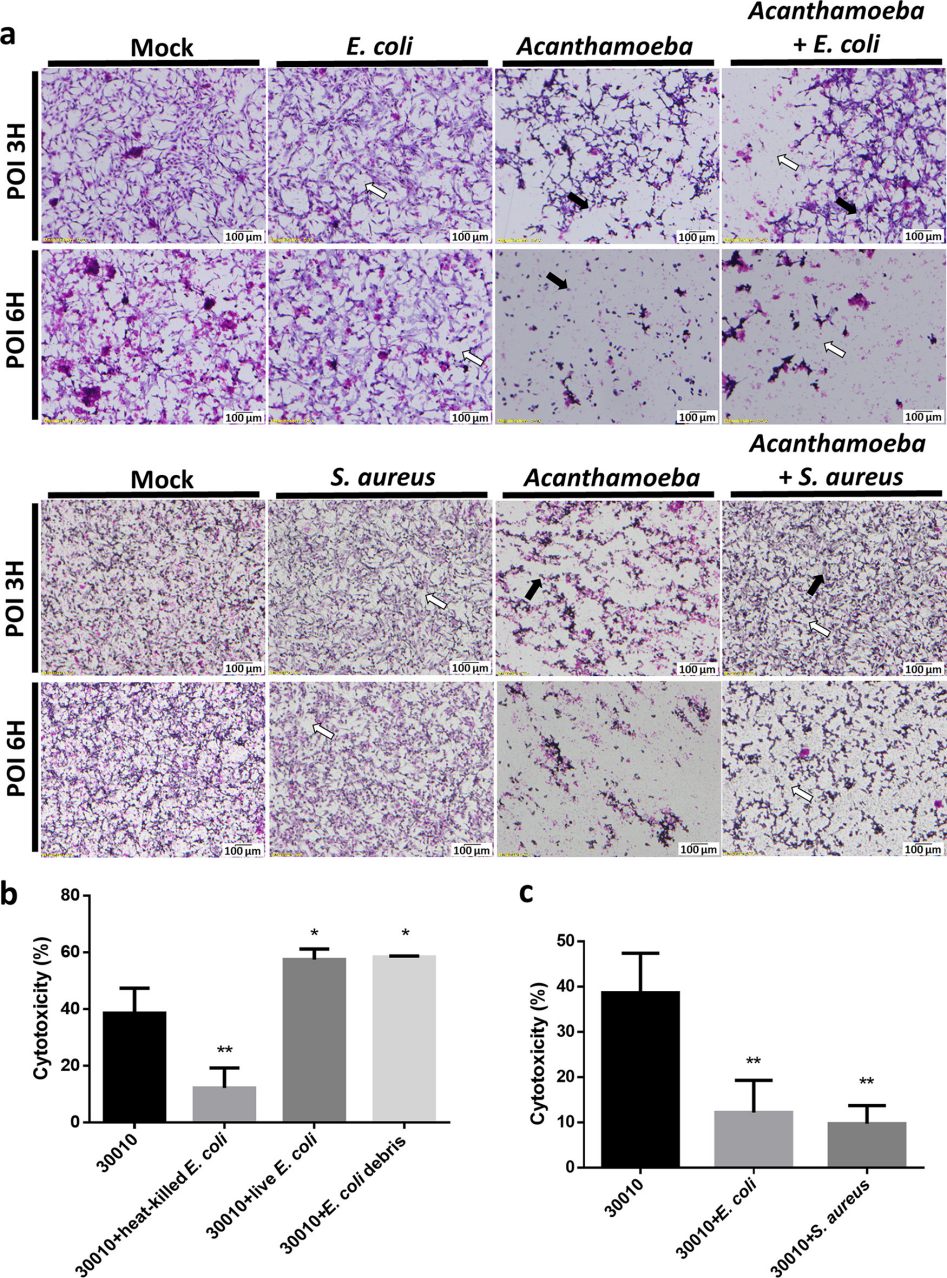
Commensal interference in the cytopathogenicity of Acanthamoeba castellanii on C6 cells. (a) Treatment of rat glial C6 cells with A. castellanii ATCC-30010 alone (black arrows) or with heat-killed Escherichia coli or Staphylococcus aureus (white arrows); evaluation of the cytopathic effects (CPE) by Giemsa staining after incubation for 3 and 6 h. (b) Lactate dehydrogenase assay after C6 cells were treated with A. castellanii ATCC-30010 alone, heat-killed E. coli, live E. coli, or E. coli debris for 6 h. The data are representative of three independent experiments. (c) Lactate dehydrogenase assay after the treatment of C6 cells with A. castellanii ATCC-30010 alone, heat-killed E. coli, or heat-killed Staphylococcus aureus for 6 h. The data are representative of three independent experiments. *, *P < *0.05; **, *P < *0.01, according to Student’s *t* test.

**FIG 2:**
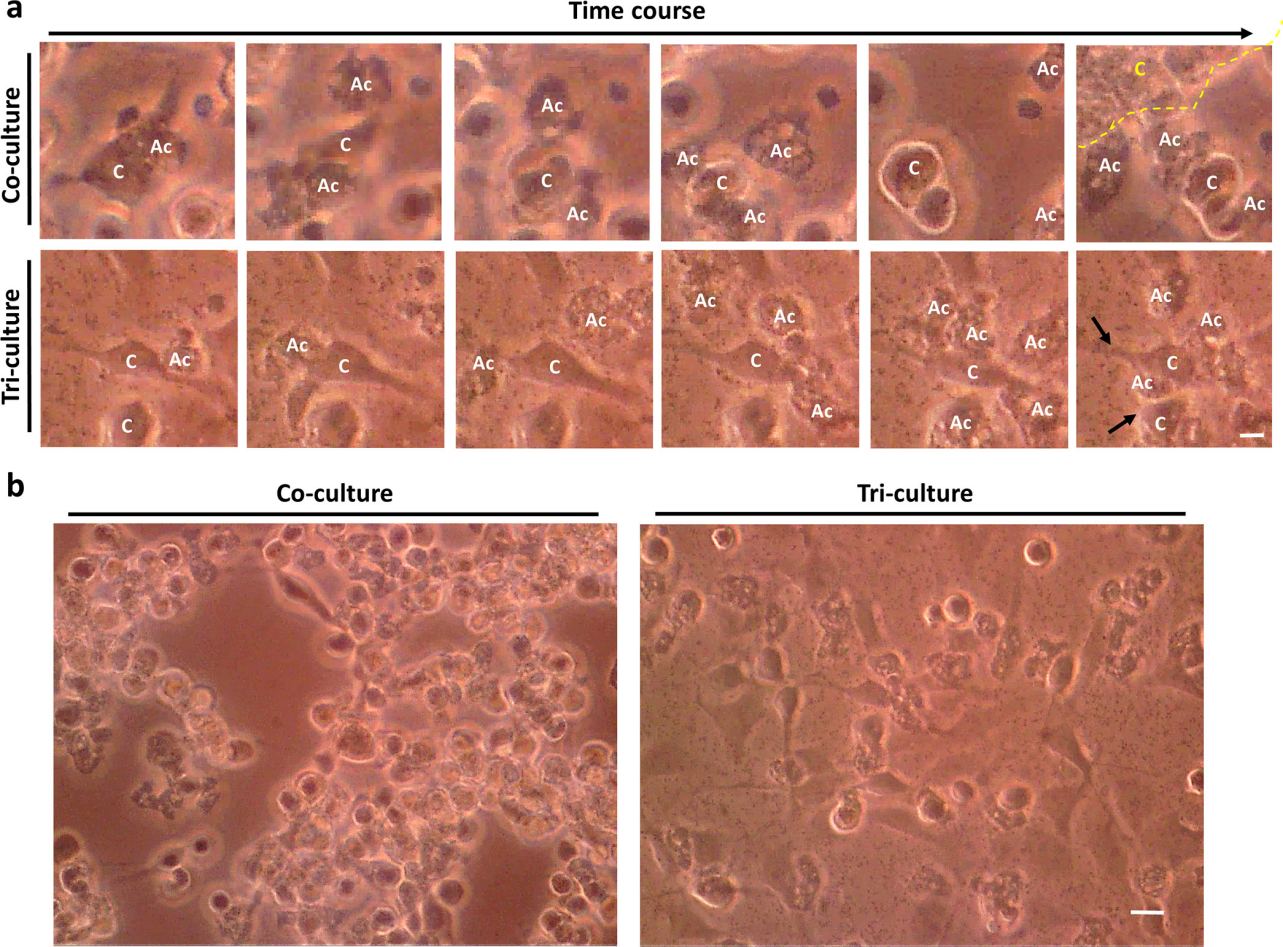
Time course of C6 cells cocultured/tricultured with Acanthamoeba castellanii or A. castellanii plus Escherichia coli within 6 h. (a) Time course micrographs show the cytopathic effects (CPE) of C6 cells induced by being crawled over by A. castellanii cells and interference due to bacterial presence. The yellow dotted line encircles clusters of detached cells after A. castellanii cells have crawled over them. The black arrows indicate extensive attachment of the cells owing to bacterial presence. C, C6 cells; Ac, A. castellanii. (b) Light micrographs showing the CPE of C6 cells after treatment with A. castellanii or A. castellanii plus E. coli. Bar = 25 μm.

To understand the effects of commensals on the pathogenesis of Acanthamoeba castellanii, we assessed the CPE on cells by coculturing A. castellanii with Escherichia coli or Staphylococcus aureus. Cell monolayers showed different disruption levels at 3 h postinfection (POI), and heat-killed commensals inhibited the ameba’s pathogenesis within 6 h POI (Fig. 1a). We further quantified the cytotoxicity of A. castellanii in the presence of commensal coculture using the lactate dehydrogenase (LDH) assay. Coculture of A. castellanii with heat-killed and intact E. coli reduced its cytotoxicity, but this did not occur with live E. coli and bacterial debris (Fig. 1b). S. aureus, a Gram-positive bacterium, also showed similar effects on the cytotoxicity of A. castellanii in the LDH assay (Fig. 1c). To validate these results, we performed coculture and triculture studies. As shown in Figure 2, A. castellanii cells repeatedly crawled over nearby mammalian cells, resulting in cell rounding in coculture. In contrast, A. castellanii cells in triculture showed active feeding behavior around heat-killed commensals and ingested them (Fig. 2). These findings suggest that intact commensals might play a crucial role in the ameba’s pathogenicity.

### The presence of commensals interfered with the phagocytic ability of *Acanthamoeba*.

**FIG 3:**
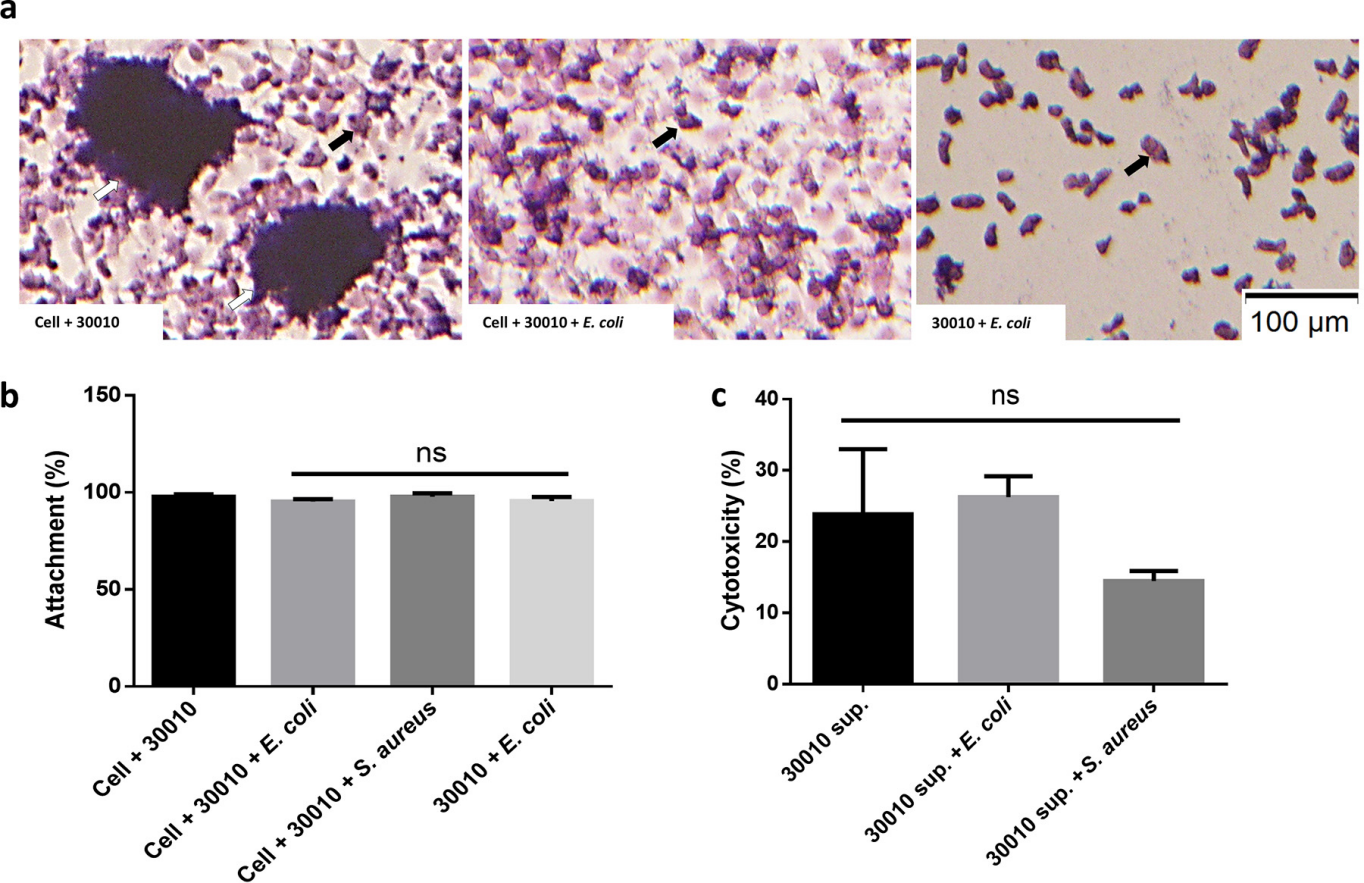
Acanthamoeba castellanii cell adhesion and protein secretion ability in the presence of commensals. (a) Images showing A. castellanii (black arrows) either attaching to the surface in the presence of bacteria or not. The dead cells were aggregated (white arrows) under A. castellanii invasion without bacteria. (b) Attached A. castellanii cells were counted after 6 h of coculture/triculture with bacteria or with C6 cells plus bacteria. The data are representative of three independent experiments. (c) Supernatants (sup) containing the proteins secreted by A. castellanii cells were added to C6 cells or C6 cells plus commensal bacteria. The data are representative of three independent experiments. ns, not significant according to Student’s *t* test, compared to the attachment rate and cytotoxicity.

**FIG 4:**
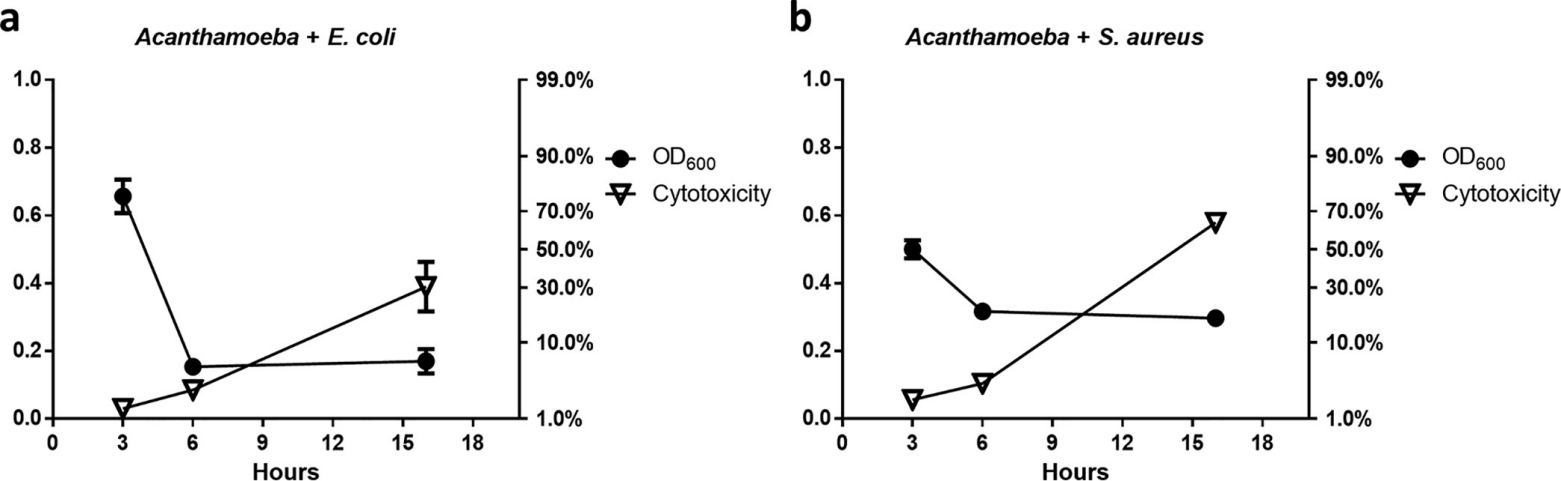
Lactate dehydrogenase assay and measurement of the concentration of heat-killed commensal bacteria to assess their interference in Acanthamoeba castellanii phagocytosis. C6 cells were tricultured with A. castellanii and an initial concentration at OD_600_ = 0.6 of heat-killed Escherichia coli (a) or Staphylococcus aureus (b) cells. The lactate dehydrogenase produced by the dead cells and the concentration of the commensals were measured at 3, 6, and 16 h. The data are representative of three independent experiments. The data are expressed as the mean ± standard deviation (SD).

A. castellanii pathogenicity occurs via three major mechanisms: adhesion, protein secretion, and phagocytosis. We investigated whether commensals interfered with the ameba’s pathogenesis mechanisms. The CPE assay showed that the presence or absence of heat-killed commensals did not inhibit the attachment of A. castellanii cells (Fig. 3a). The percentage of attached A. castellanii cells was approximately 100% in all groups (Fig. 3b). Furthermore, culture supernatant suspected of having secreted proteins of A. castellanii, along with heat-killed commensals, was added to the C6 cells. However, the commensals did not neutralize the cytotoxicity of the secreted proteins (Fig. 3c). We further tested the effect of commensals on the phagocytic ability of A. castellanii by comparing the bacterial concentration with A. castellanii cytotoxicity and found them to be inversely proportional within 6 h POI. However, at 8 h POI, the concentration of commensals remained constant and A. castellanii cytotoxicity was elevated (Fig. 4), which indicated that the presence of commensals interfered with A. castellanii phagocytosis, resulting in cytotoxicity reduction.

### Preservation of cell junctions by commensals.

**FIG 5:**
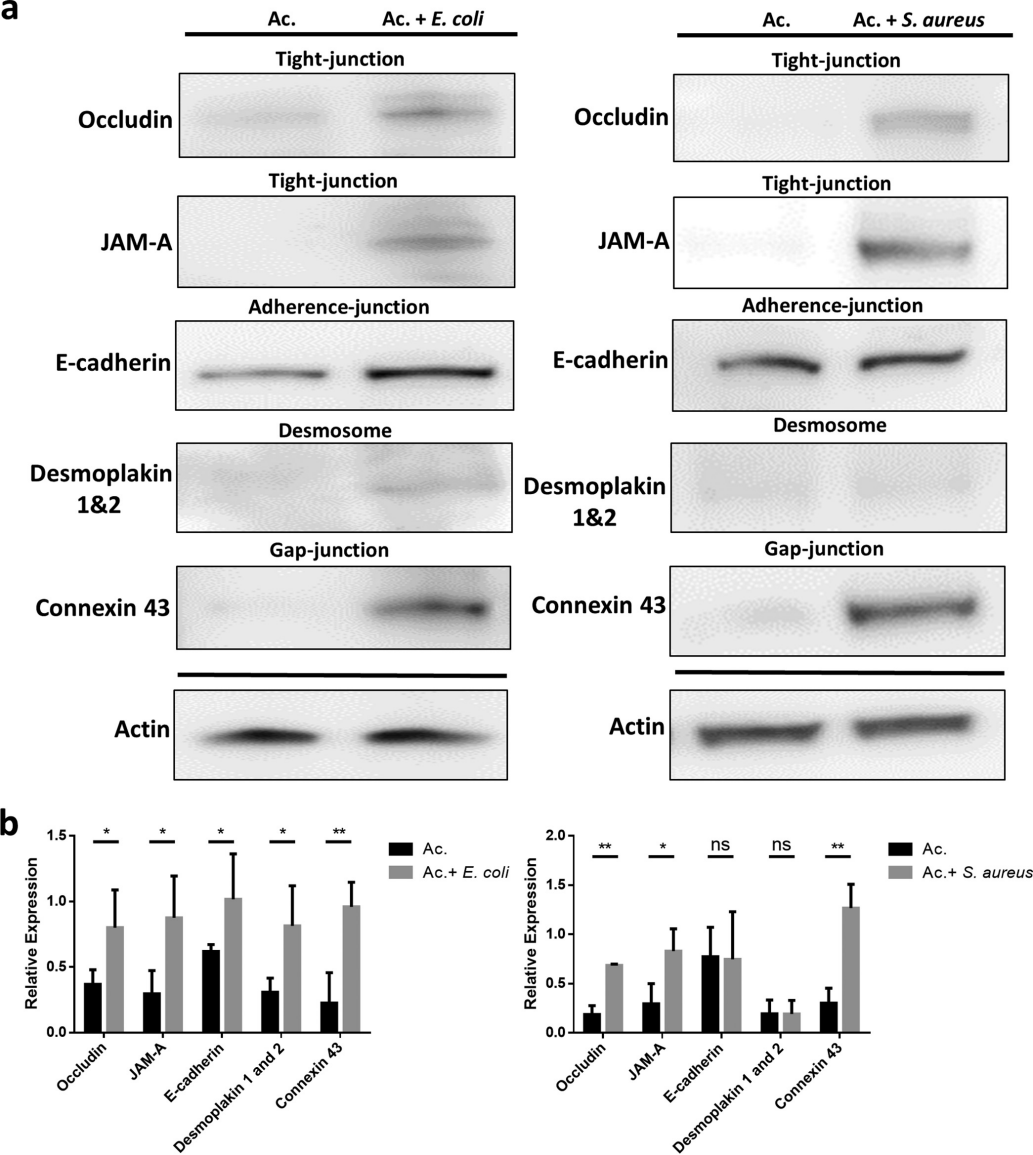
Protection of the cell junctions in the presence of commensals. (a) Sodium dodecyl sulfate-polyacrylamide gel electrophoresis (SDS-PAGE) showing the expression level of tight junction (occludin, JAM-A), adherence junction (E-cadherin), desmosome (desmoplakin 1 and 2), and gap junction (connexin 43) proteins in epithelium A549 coculture/triculture with Acanthamoeba castellanii cells alone or with Escherichia coli or Staphylococcus aureus cells. Examination of the whole-cell extracts by Western blot analysis after 6 h of treatment, with actin used as a loading control. (b) Quantification of the cell junction relative expression level in epithelium A549 coculture/triculture with A. castellanii cells alone or with E. coli or S. aureus cells. The data are representative of three independent experiments, and the protein expression strength was quantified using ImageJ software. *, *P < *0.05; **, *P < *0.01; ns, not significant according to Student’s *t* test. Ac., *Acanthamoeba*.

The disruption of cell junctions is a crucial step for the invasion and ultimately pathogenicity of A. castellanii. Western blotting was performed on A549 epithelial cells to screen for the preservation of common cell junctions on epithelial cells, including tight junctions, adherence junctions, desmosomes, and gap junctions. Interestingly, the presence of heat-killed E. coli cells significantly reduced the relative expression of epithelial cell junctions, and heat-killed S. aureus cells reduced the levels of occludin, junctional adhesion molecule A (JAM-A), and connexin 43 (Fig. 5). This suggests that cell junctions were protected by the presence of commensals, which further prevented the cells from rounding and dying. Thus, the absence of commensals would have led to the breakdown of cell junctions, leading to A. castellanii penetration.

### The integrity of the corneal epithelium is preserved by commensals during *Acanthamoeba* invasion.

**FIG 6:**
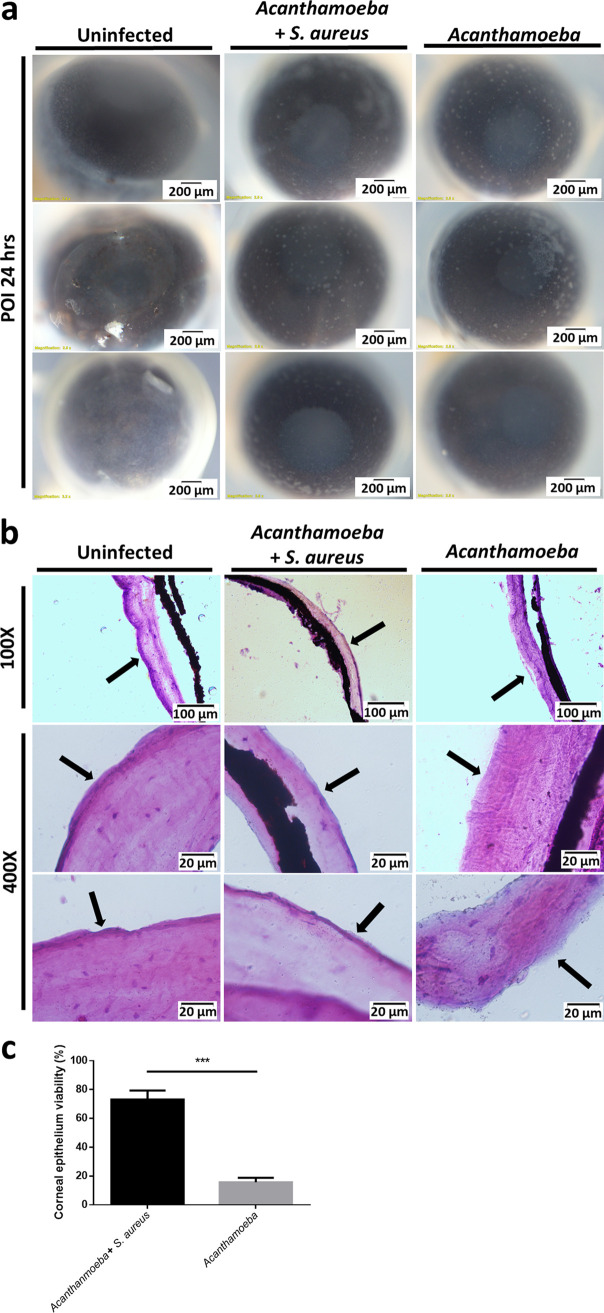
Presence of commensals during Acanthamoeba castellanii invasion in the eyeballs of an *ex vivo* mouse model. (a) *Ex vivo* images showing different conditions of the corneal epithelial cells peeling under A. castellanii invasion alone or in the presence of commensal bacteria at 24 h postinfection. (b) Biopsy section after hematoxylin and eosin (H&E) staining reveals the cell layer completeness (black arrows) after A. castellanii invasion alone or in the presence of commensals. (c) Quantification of the corneal epithelium viability in an *ex vivo* model. The data are representative of three independent experiments. ***, *P < *0.001, according to Student’s *t* test.

The *ex vivo* mouse model was used to test the effect of commensal bacteria on corneal epithelium abrasion by A. castellanii. Before infection, the eyeballs of all mice groups had a transparent appearance owing to a preserved corneal epithelium and good refraction. Several scrapes appeared on the ocular surface 24 h POI with A. castellanii alone. However, in the group subjected to A. castellanii infection along with heat-killed commensals, only a few scrapes were noted (Fig. 6a). A biopsy was performed to evaluate the level of cell damage using hematoxylin and eosin (H&E) staining. The outer layer of the corneal epithelium was deeply stained, with a clear boundary, when no infection was present, which was also observable for A. castellanii invasion with bacteria. However, for A. castellanii infection alone, there was no intact deep-stained corneal epithelium (Fig. 6b). The average corneal thickness of the mouse eyeballs was 11.06 μm. However, the eyeballs treated with *Acanthamoeba* only presented 15.70% corneal epithelium viability and had an average thickness of 1.74 μm. In contrast, the eyeballs with *Acanthamoeba* invasion with bacteria retained 73.16% of the corneal epithelium viability and had an average thickness of 8.10 μm (Fig. 6c). Thus, the *ex vivo* mouse model provided evidence for the crucial role of commensals in mitigating A. castellanii pathogenicity.

## DISCUSSION

As a free-living protozoan, A. castellanii usually ingests bacteria by directional movement and digests them through phagocytosis (27). A previous study demonstrated that A. castellanii pseudopodia contain several receptors that attach to the targets and ingest them (28, 29). However, A. castellanii motility decreases under sufficient nutrient conditions (30, 31), suggesting that it can also feed at a fixed position without motility and hunting. Interestingly, our CPE assay and time course capture data were consistent with previous findings (32). The presence of intact commensals significantly reduced A. castellanii cytotoxicity. Although A. castellanii still crawled on the epithelium, the cell damage was reduced. These data show that if individuals have a healthy microbiota, the incidence of diseases caused by A. castellanii, like AK and GAE, will be lower.

A. castellanii uses adhesion as the initial step during the onset of its invasion (33). Proteolytic enzymes and proteases secreted by A. castellanii, including cysteine proteases, serine proteases, and metalloproteases (34–36), catalyze the degradation of extracellular peptide bonds (37) and stimulate apoptosis in neuroblastoma cells (38). Additionally, phagocytosis by A. castellanii involves the engulfment of host epithelial cell debris and small particles, such as bacteria and yeast (39). Based on this information, we examined the effect of commensals on each step that influenced pathogenesis. Evaluation of A. castellanii attachment and secreted proteins indicated that commensals did not affect the cytotoxicity of either factor to mammalian cells. In contrast, a sufficient concentration of commensals significantly reduced the cytotoxicity caused by A. castellanii phagocytosis. Furthermore, upon the depletion of commensal bacteria, the cytotoxicity of A. castellanii increased. This suggests that commensals obstructed the phagocytic ability of A. castellanii during infection.

Junction proteins are molecular components that occupy the space between the contiguous body surface of epithelial cells, which is also important for cell monolayer maintenance (40). However, once the epithelium loses these connections formed by the junction proteins, apoptosis occurs (41). In our study, quantification of the four major epithelial cell junctions showed that commensals protected these cell junctions. The *ex vivo* mouse model showed the same effects of commensal presence during A. castellanii infection. H&E staining images showed that peeling of the epithelial layer was prevented in the presence of commensals, which indicates that commensals might serve as a natural barrier to A. castellanii infection.

Thus, we found that intact Gram-positive or Gram-negative commensals significantly reduced the phagocytic activity of A. castellanii on epithelial cells. In addition, we demonstrated that commensals protected epithelial cell junctions and resulted in cell survival. Finally, we used an *ex vivo* mouse model to validate this effect. Biopsy data showed that the epithelium peeling decreased and a clear epithelial boundary was present in cases where commensals were involved in A. castellanii invasion. Future studies should investigate whether probiotic supplementation can protect humans from A. castellanii infection and maintain a natural barrier.

## MATERIALS AND METHODS

### *Acanthamoeba* culture.

The standard strain Acanthamoeba castellanii ATCC-30010 was obtained from the ATCC (Manassas, VA, USA). A. castellanii strains were cultured in protease peptone-yeast extract-glucose (PYG) medium (20 g peptone, 2 g yeast extract, and 18 g glucose; 1 g sodium citrate dehydrate, 0.98 g MgSO_4_ × 7H_2_O, 0.355 g Na_2_HPO_4_ × 7H_2_O, 0.34 g KH_2_PO_4_, and 0.02 g Fe[NH_4_]_2_[SO_4_]_2_ × 6H_2_O, pH 6.5, in 1,000 mL distilled water and autoclaved at 121°C for 15 min) (42) at 28°C in cell culture flasks and maintained after washing in Page’s modified Neff’s ameba saline (PAS; 1.2 g NaCl, 0.04 g MgSO_4_–7H_2_O, 0.03 g CaCl2, 1.42 g Na_2_HPO_4_, and 1.36 g KH_2_PO_4_ in 1 L double-distilled water [ddH_2_O]) (43).

### Cell culture.

Glioma C6 and non-small cell lung cancer cells (A549) were cultured in Dulbecco’s minimum essential medium (DMEM) supplemented with 10% fetal bovine serum (FBS) and pen-strep (100 U/mL penicillin and 100 μg/mL streptomycin) (44). Cells were maintained at 37°C and 5% CO_2_.

### Bacterium processing.

Escherichia coli strain K-12 and methicillin-resistant Staphylococcus aureus cells were used as the ocular microbiota. Heat-killed microbes were subjected to heat shock at 90°C for 5 min, similarly to our previous study (45). To acquire intact microbes, the bacterial debris was collected from live microbes sonicated in DMEM using an ultrasonic probe (BO3 ultrasonic processor UP 1200; Cromtech India). In addition, the optical density (OD) of the processed microbes was adjusted to OD_600_ = 0.5 before supplementation.

### Adhesion assays.

A. castellanii cells were cultured in PYG medium (pH 6.5) at 28°C, and the medium was refreshed 15 to 20 h before the experiments. The trophozoite form of A. castellanii was screened by microscopy and collected by placing the flasks on ice for 30 min with gentle agitation. The detached cells were collected by centrifugation at 3,000 rpm for 5 min. Then, they were suspended in 200 ml Page’s modified Neff’s ameba saline (PAS) and seeded at 1 × 10^5^ amebae/well and incubated with heat-killed E. coli strain K-12 or MRSA (OD_600_ = 0.5) for 1 h at 28°C. The unattached amebae were gently collected, added to PAS, and stained with trypan blue for hemocytometer counting. The attachment rate was calculated as follows: ([total amebae – unattached amebae]/total amebae) × 100 = % attachment (46). All measurements were repeated thrice.

### Analysis of neutralization of the secreted proteins.

A. castellanii strains were seeded into 10-cm culture dishes and cultured in PYG medium (pH 6.5) at 28°C. After reaching confluence, the culture medium was replaced with PAS and cultured for 4 h. The PAS medium from the A. castellanii culture was collected and supplied to the cultured C6 cells. Simultaneously, the heat-killed Escherichia coli strain K-12 or methicillin-resistant Staphylococcus aureus cells were added to the C6 cells along with the medium. Analysis of the neutralization of the secreted proteins and their cytopathic effect was performed after 4 h of coincubation (47).

### Cytopathic effect analysis.

C6 cells were cultured in 6-well plates (Falcon Plastics; no. 38016), and on attaining confluent monolayers, the medium in the wells was replaced with serum-free 20% DMEM before treatment. A. castellanii cells, alone or with heat-killed E. coli K-12/MRSA cells, were added to the culture medium and incubated for 6 h. After incubation, the cell monolayers were fixed with 2% paraformaldehyde and stained with Giemsa stain (Merck, Darmstadt, Germany) (48).

### Lactate dehydrogenase assay.

Cells were grown overnight in 24-well plates to obtain monolayers. These cell monolayers in serum-free 20% DMEM medium were incubated with A. castellanii (1 × 10^5^ amebae/well) at 37°C in a 5% CO_2_ incubator for 6 h. Cell supernatants were collected, and the cytotoxicity was determined by measuring the LDH release (cytotoxicity detection kit; Roche). The absorbance of the product obtained from the reaction with LDH was measured at 492 nm using a Multiskan SkyHigh microplate spectrophotometer (Thermo Fisher Scientific). For the positive control, 1% Triton X-100 was used on the cell monolayers for 100% cell death. In contrast, untreated cells served as the negative control. The absorbance was converted to cytotoxicity as follows: ([sample value – negative-control value]/[positive-control value – negative-control value]) × 100 = % cytotoxicity (49). All the determined CPE values were repeated thrice.

### Time-course and image capture.

Dynamic images of A. castellanii invasion were captured using an Olympus microscope (Tokyo, Japan) with a digital camera (AM7025X Edge; Dino-Lite, Taiwan). The capture interval of each image was set at 10 min using DinoCapture version 2.0 (Dino‐Lite). To characterize the influence of the commensal bacteria, coculture, and triculture images were captured using an MV PLAPO 2XC dissection microscope (Olympus) after methanol fixation, Giemsa staining, and evaluation of the *ex vivo* mouse model.

### Western blotting.

A549 cells were cultured in 10-cm petri dishes and grown at 37°C in a 5% CO_2_ incubator in DMEM containing FBS. After 24 h, the A549 cell monolayer was tricultured with A. castellanii cells (1 × 10^5^ amebae/dish) and heat-killed S. aureus/E. coli cells for 6 h. The protein lysate was collected after incubation, and the total protein extract was separated by sodium dodecyl sulfate-polyacrylamide gel electrophoresis (SDS-PAGE). The proteins were transferred onto a polyvinylidene fluoride membrane, which was blocked with 3% skimmed milk in phosphate-buffered saline at 25°C, and then incubated overnight with monoclonal antibodies at 4°C. The blots were then incubated with horseradish peroxidase (HRP)-conjugated IgG for 1 h at 25°C. Proteins were detected using Immobilon Western HRP substrate (50). The optical densities of the bands were measured using ImageJ software (51).

### Ethics statement.

All the animal experimental procedures were reviewed and approved by the Institutional Animal Care and Use Committee (IACUC) of the Laboratory Animal Research Center at National Cheng Kung University (NCKU) (approval NCKU-IACUC-109-015).

### *Ex vivo* mouse model.

Because the *in vivo* A. castellanii infection model is unstable to date, we developed an *ex vivo* mouse model modified from a previous study on *Acanthamoeba* infection (52, 53). Briefly, three eyeballs from each group of mice were obtained from sacrificed ∼6 to 8-week-old BALB/c mice without ocular disorders. The separated eyeballs were placed with the cornea side up in a 48-well plate containing 20% DMEM for 5% soft agar fixation. A. castellanii cells (1 × 10^5^ amebae) with/without heat-killed bacteria (OD_600_ = 0.5) were added to the wells and incubated at 28°C. After 24 h, the appearance of the eyeballs was recorded using a dissection microscope; they were then fixed in 10% formalin for staining with H&E solution (54). Quantification of the corneal epithelium viability was performed using ImageJ software to evaluate the corneal epithelium thickness. Briefly, biopsy specimen images were inverted to a single color. The standard corneal epithelium thickness was adjusted using the average epithelium thickness of the control eyeballs. Each group of eyeballs treated with A. castellanii with/without heat-killed bacteria was measured in the same way as the control eyeballs. The corneal epithelium viability of the cells was calculated as follows: (average thickness of single eyeball/average thickness of control) × 100%. Three independent experiments were conducted.

### Statistical analysis.

The data were expressed as the mean ± standard deviation (SD). All comparisons were analyzed using unpaired two-tailed Student’s *t* tests. Statistical significance was set at *P* < 0.05. The statistical data were calculated and analyzed using GraphPad Prism version 5.0 software (La Jolla, CA, USA).

### Data availability.

The raw data supporting the conclusions of this article will be made available by the authors upon reasonable request.
